# Prevalence and variation of Chronic Kidney Disease in the Irish health system: initial findings from the National Kidney Disease Surveillance Programme

**DOI:** 10.1186/1471-2369-15-185

**Published:** 2014-11-25

**Authors:** Austin G Stack, Liam F Casserly, Cornelius J Cronin, Tetyana Chernenko, Walter Cullen, Ailish Hannigan, Rajiv Saran, Howard Johnson, Gemma Browne, John P Ferguson

**Affiliations:** Departments of Nephrology and Internal Medicine, University Hospital Limerick, Limerick, Ireland; Graduate Entry Medical School, University of Limerick, Limerick, Ireland; Kidney Epidemiology and Cost Center, School of Public Health, University of Michigan, Ann Arbor, MI USA; Health Intelligence Unit, Health Services Executive, Dublin, Ireland; Department of Epidemiology and Public Health, University College Cork, Cork, Ireland; Department of Medicine, Clinical Academic Liaison Building, St Nessans Rd, Limerick, Ireland

**Keywords:** CKD surveillance, Health system, Epidemiology, Risk factors

## Abstract

**Background:**

Chronic Kidney Disease (CKD) is a major non-communicable chronic disease that is associated with adverse clinical and economic outcomes. Passive surveillance systems are likely to improve efforts for prevention of chronic kidney disease (CKD) and inform national service planning. This study was conducted to determine the overall prevalence of CKD in the Irish health system, assess period trends and explore patterns of variation as part of a novel surveillance initiative.

**Methods:**

We identified 207, 336 adult patients, age 18 and over, with serum creatinine measurements recorded from a provincial database between 2005-2011 in the Northwest of Ireland. Estimated glomerular filtration rates (eGFR) were determined using the Chronic Kidney Disease Epidemiology Collaboration (CKD-EPI) equation from standardized creatinine measurements and the presence of CKD was defined as eGFR <60 ml/min per 1.73 m^2^. Age and sex-specific prevalence estimates were determined for each group while generalized estimating equations (GEE) and multivariable logistic regression were used to explore associations using adjusted odds ratios (AOR) and 95% confidence intervals (95% CI).

**Results:**

The prevalence of CKD in the health system was 11.8% (95% CI 11.8-12.1); 10.9% in men (10.7-11.1) and 12.6% in women (12.4-12.8). This corresponded to a detection rate of 4.5% (5.1% in women and 3.9% in men). The prevalence of CKD was significantly higher in women than in men (12.6% versus 10.9%, P < 0.001), older age groups, and among patients with a history of Acute Kidney Injury (AKI) than without (45.2% versus 10.7%, P < 0.0001). Multivariable analysis identified advancing age, female gender, location of medical supervision, county of residence, and AKI as significant determinants of prevalence.

**Conclusion:**

The prevalence of CKD in the Irish health system is 11.8% corresponding to a detection rate of 4.5% in the general population. Demographic, geographic factors and acute kidney injury episodes are important determinants of disease burden. Passive surveillance of CKD is both feasible and desirable within the Irish health system, and offers huge opportunities for targeted prevention programmes and improved clinical outcomes.

**Electronic supplementary material:**

The online version of this article (doi:10.1186/1471-2369-15-185) contains supplementary material, which is available to authorized users.

## Background

Chronic Kidney disease (CKD) has emerged as a major public health epidemic, which contributes substantially to adverse clinical and economic outcomes [1–4]. It is estimated that at least 1 in 10 individuals of the general population have some degree of kidney impairment and have substantially increased risk for death, even prior to developing end-stage kidney disease (ESKD) [1–3]. For those who develop ESKD, the outcomes are even poorer with an average life expectancy of <5 years [5–8]. For many patients, CKD is largely an asymptomatic disease that can progress silently without treatment for many patients unless it is actively screened for by medical providers. Early detection of CKD and the application of specific interventions provide major opportunities to prevent and delay progression [9]. Targeted surveillance systems are now seen as essential platforms to combat the rising tide of CKD and its attendant sequelae [10–15].

Although the epidemiology of CKD is well characterized in many countries, there remain several unanswered questions. The extent to which CKD is captured within existing health systems has not been fully explored and is a crucial starting point for most surveillance programmes. A detailed exploration of differences in CKD prevalence by geography is an equally important goal to uncover possible differences in risk factor burden and disparities in clinical care. Acute Kidney Injury (AKI) is a common illness, with reported prevalence of up to 7.1% of all hospital admissions [16]. Emerging evidence suggests that AKI events accelerate the risk of developing CKD [17], yet its impact on CKD within an Irish population has not yet been studied. Finally, the location of medical supervision where cases are first identified by physician providers is an important consideration as it provides opportunities for early initiation of prevention strategies. In Ireland, the lack of robust surveillance systems for tracking and monitoring CKD burden and outcomes has been identified a major deficiency. The 2007 Survey of Lifestyle, Attitudes and Nutrition (SLÁN), a nationally representative sample of all subjects’ age >45, has provided the first national estimate of CKD prevalence in Ireland with a figure of 11.6% [18]. This important study, however; was restricted to subjects >45 years, unable to assess longitudinal trends, and did not measure the extent to which CKD was captured within the Irish health system.

To overcome these deficits, we have initiated a programme for CKD surveillance in Ireland to improve our knowledge. The major objectives of this study were to 1) describe the prevalence of CKD within the health system, 2) explore patterns of variation according to demographic and geographic characteristics, and location of medical supervision, and to 3) assess the impact of AKI on disease prevalence. These objectives were pursued by creating a passive CKD surveillance system using routinely collected data from a regional health system.

## Methods

We conducted a retrospective observational study of all patients with measured serum creatinine concentrations from a regional laboratory information system in the Northwest of Ireland. The laboratory system captured all blood chemistries from inpatient admissions and outpatient attendances at two regional hospitals and as well as primary care practices across the region. Ethical approval for the study was granted by the Ethics Committees at Sligo and Letterkenny General Hospitals.

### Sample

From January 1^st^ 2005 to 31^st^ December 2011, a total of 278,630 patients underwent 69,594,271 laboratory test evaluations in the Northwest region. The current analysis was restricted to adult participants, 18 years of age or older with recorded serum creatinine measurements and non-missing data on sex (n = 206, 729). Creatinine tests administered during periods of dialysis and AKI were excluded. Estimated glomerular filtration rate (eGFR) in ml/min per 1.73 m^2^ was determined for patients using the Chronic Kidney Disease Epidemiology Collaboration (CKD-EPI) and the Modification of Diet in Renal Disease Study (MDRD) [19, 20].

### Data collection

Patient and laboratory information systems captured data on demographic factors, county of residence, location of supervision, primary location of blood draw, and dialysis indicator variables. Serum creatinine was measured using the modified kinetic Jaffe method and creatinine values were calibrated to be traceable to an isotope dilution mass spectrometry (IDMS) reference measurement procedure to ensure standardization. Chronic Kidney Disease was defined according to the Kidney Disease Dialysis Quality Outcome Initiative (KDOQI) guidelines based on eGFR measurements expressed in ml/min/1.73 m^2^ and categorized as Stage 3 (eGFR <30-59), Stage 4 (eGFR 15-29 and Stage 5 (eGFR <15). Higher categories of eGFR were categorized as normal (eGFR >90) or mildly reduced (eGFR 60- 89) [21]. The presence of Acute Kidney Injury (AKI) was defined by the Kidney Disease Improving Global Outcomes (KDIGO) criteria [16]. The location of medical supervision was defined as the location where the serum creatinine test was first ordered by the supervising health professional and categorised as; inpatient location (IP), outpatient department (OP), general practice (GP), and outside community facility (OS). The identification of these locations was considered important as we surmised that testing rates for CKD would vary by clinical setting. County of residence for each patient was extracted from the patient administration system and allowed us to classify patients by geography. The principal counties served by the regional database included county Donegal, Sligo and Leitrim and these accounted for 95.5% of all laboratory testing.

### Estimation of Chronic Kidney Disease prevalence

*The prevalence of CKD in the health system* for a calendar year was defined as percentage of patients with a mean eGFR <60 ml/min/1.73 m^2^ with corresponding 95% Confidence Intervals (CI). For patients with more than one serum creatinine value in a calendar year, the corresponding eGFR levels were estimated and the means of all eGFR values were calculated. Creatinine test results that satisfied criteria for the diagnosis of AKI based on the KDIGO criteria were excluded from prevalence estimation. T*he prevalence of detectable CKD within the Northwest population* was derived using national census data for 2006 and 2011 with projected estimates for the intervening years [22]. The prevalence of detectable CKD in the population, stratified by age and sex categories, was defined as the ratio of the number of patients with a mean eGFR <60 ml/min/1.73 m^2^ (numerator), restricted to that group of patients known to reside in either Leitrim, Donegal or Sligo, to the combined population of Sligo, Leitrim and Donegal for a calendar year (denominator).

### Statistical analysis

Generalized Estimating Equations (GEE) and multivariable logistic regression models were fitted to explore the associations of demographic, clinical and geographic factors with CKD prevalence. In each model, the response variable was eGFR <60 ml/min/1.73 m^2^. The explanatory variables included age modeled in categories, sex, county of residence, location of medical supervision, presence and frequency of AKI and calendar year. A final multivariable model was constructed to explore the relative contribution of all factors with CKD prevalence. A working independence correlation structure was assumed when fitting the model so that the final parameter estimates for each model agree with the corresponding logistic regression. The sandwich estimator was used to adjust the standard errors of the estimated coefficients to account for the longitudinal aspect of the design. The associations of explanatory factors with CKD presence were represented by adjusted odds-ratios (AOR) and 95% CI. Wald statistics from the corresponding generalized estimating equations were used to test for the significance of these associations. Finally Joinpoint regression analysis [23] was used to assess prevalence trends in health system and the population. The Joinpoint model used allowed for heteroskedastic standard errors in the prevalence estimates and an autoregressive dependence structure to reflect the fact that the same patients may contribute to the prevalence estimates in successive years. Several sensitivity analyses were conducted to explore the robustness of our observations. First, we compared prevalence estimates using both the MDRD and CKD-EPI equations. Second, we also considered the minimum, median and maximum eGFR values over a calendar year as additional summary measures for a particular patient in estimating of prevalence. The final analytic dataset was constructed using R software and statistical analysis was performed using SAS v9.3 and Joinpoint Regression Program [24].

## Results

### Baseline characteristics of the population

The characteristics of patients within the Northwest Heath System are described in Table 1. The mean age was 54.2 (±18) years, 54.1% were female and 41.3% were over the age of 60 years. The majority of patients were resident in the counties of Donegal (53.5%), Sligo (29.6%) and Leitrim (12.4%), with smaller proportion of patients (4.51%) living in other counties (see Table 1 for details). Testing for kidney function was ordered by general practitioners (GP) for 67.8% of patients and by emergency physicians (EP) for 15% of patients. The average creatinine concentration (μmol/L), and estimated GFR (ml/min/1.73 m^2^) for the entire population were 80.1 (±28.8) and 86.8 (±22.7) respectively. Overall, 3.2% of patients within the health system had evidence of AKI, and this was significantly higher for patients with CKD than those without (12.6% versus 2.0% respectively, P <0.001).

**Table 1:** **Characteristics of patients in the Irish health system** ^**1**^

Patient characteristics	Entire cohort	2005	2006	2007	2008	2009	2010	2011
**Count (n)**	206,729	64,674	71,118	75,956	79,300	81,338	85,200	89,679
**Demographics**								
Age	54.2 (18.2)	54.4 (18.4)	54 (18.4)	53.9 (18.4)	54.1 (18.2)	54.2 (18.2)	54.4 (18.1)	54.4 (18.1)
**Sex**								
% Women	54.1	54.6	54.0	54.3	54.4	54.2	53.6	53.9
% Men	45.9	45.4	46.0	45.7	45.6	45.8	46.4	46.1
**County of Residence (%)**								
Donegal	53.5	52.9	52.3	53.2	53.6	53.9	54.0	54.5
Sligo	29.6	30.5	30.8	29.9	29.6	29.5	29.0	28.4
Leitrim	12.4	12.0	12.2	12.4	12.3	12.3	12.6	12.6
Other County^5^	4.51	4.60	4.69	4.51	4.42	4.33	4.47	4.56
**Location of Supervision (%)** ^**2**^								
Outpatient Department	7.1	5.3	4.5	4	7.8	8.7	8.7	9.5
General Practitioner	67.8	64.3	67.4	68.2	67.7	67.6	69.3	69.4
Emergency Room	15.1	17.3	16.3	16	14.4	14.5	14.1	14
Inpatient Location	5.0	7.5	6.5	6.5	4.7	4.2	3.3	3.1
Outside facility	5.0	5.6	5.2	5.3	5.5	5.0	4.7	4.0
**Kidney Function** ()								
Serum creatinine count	1,388,625	171,922	183,565	199,533	205,493	203,938	204,204	219,970
Serum creatinine (μmol/L)	80.1 (28.8)	82.5 (33.4)	81 (31)	79.7 (29.2)	78.6 (28)	80 (27.3)	79.4 (26.6)	80.1 (26.8)
Mean eGFR (ml/min/1.73 m^2^)^3^	86.8 (22.7)	84.8 (23.2)	86.4 (23.1)	87.5 (22.9)	88.2 (22.7)	86.7 (22.5)	87.2 (22.3)	86.5 (22.4)
Minimum GFR (ml/min/1.73 m^2^)^3^ (ml/min/1.73 m)^2^	84.2 (23.9)	82.0 (24.5)	83.7 (24.4)	84.8 (24.3)	85.6 (24)	84.0 (23.7)	84.7 (23.4)	84.0 (23.5)
Maximum eGFR (ml/min.73 m^2^)^3^	89.2 (22.4)	87.4 (22.9)	88.9 (22.8)	90.0 (22.5)	90.6 (22.3)	89.1 (22.2)	89.4 (22)	88.8 (22.1)
**Serum Electrolytes** ()								
Sodium (mmol/L)	140.2 ( 2.8)	140.1 (2.9)	140.3 (2.8)	140.2 (2.8)	140.3 (2.8)	139.9 (2.7)	140.2 (2.7)	140.5 (2.6)
Potassium (mmol/L)	4.5 ( 0.5)	4.4 (0.5)	4.5 (0.5)	4.5 (0.5)	4.5 (0.5)	4.5 (0.5)	4.5 (0.5)	4.5 (0.5)
Urea (mmol/L)	6.2 ( 2.7)	6.1 (2.8)	6.1 (2.7)	6.1 (2.7)	6.3 (2.7)	6.3 (2.7)	6.3 (2.6)	6.1 (2.6)
Calcium (mmol/L)	2.3 ( 0.1)	2.4 (0.1)	2.3 (0.1)	2.3 (0.1)	2.3 (0.1)	2.3 (0.1)	2.3 (0.1)	2.3 (0.1)
Phosphorous (mmol/L)	1.1 ( 0.2)	1.1 (0.2)	1.1 (0.2)	1.2 (0.2)	1.1 (0.2)	1.1 (0.2)	1.1 (0.2)	1.1 (0.2)
**Hematology Variables**								
Haemoglobin (g/dl)	13.8 (1.6)	13.8 (1.6)	13.9 (1.6)	13.9 (1.6)	13.7 (1.6)	13.7 (1.6)	13.7 (1.6)	13.8 (1.6)
Serum ferritin ng/ml	130.9 (286.4)	127.3 (295.1)	124.0 (341.6)	127.8 (236.8)	128.6 (228)	135.8 (396.3)	132.7 (223.2)	137.2 (254.2)
**Other Laboratory Variables**								
Serum uric acid (UA) (*μ*mol/L)	355.7(109.5)	369.4(119.6)	352.7 (111.8)	346.8 (106.5)	352.2 (106.5)	355.7 (108.9)	354.5 (106.5)	362.9 (108.9)
**Acute Kidney Injury** ^**4**^								
% with AKI	3.2	3.3	3.5	3.4	3.2	3.1	2.8	3.0
% of AKI in patients with no CKD	2.0	1.9	2.2	2.11	1.99	2.0	1.8	1.9
% of AKI in patients with CKD	12.6	12.0	12.9	13.68	13.41	12.2	12.0	12.1

^1^Values are reported as % or mean with standard deviation.

^2^Location of Supervision is defined as the type of facility where the first test was carried out.

^3^eGFR CKD-EPI: Glomerular filtration rate (ml/min per 1.73 m^2^) was based on the Chronic Kidney Disease Collaborative (CKD-EPI) [21].

^4^The occurrence of acute kidney injury (AKI) was based on the KDIGO Definition [16].

^5^The list of counties and their relative contributions were as follows.

Donegal (n = 101,587), Sligo (n = 50,264), Leitrim (n = 22,356), Galway (n = 329), Derry (n = 418), Cavan (n = 1,251), Tyrone (n = 319), Roscommon (n = 2,403), Mayo (n = 5,546), Fermanagh (n = 158), Longford (n = 229), Westmeath (n = 15). Information on home address was unrecorded for 21,854 patients.

### Prevalence of CKD in the Irish Health System

**Figure 1:**
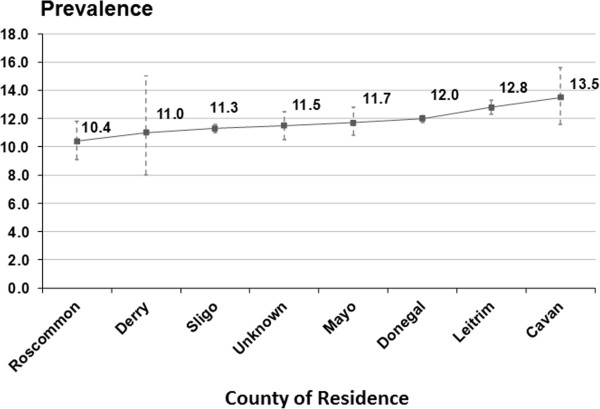
**Prevalence of CKD and 95% confidence intervals by county in the health system.** ^1^The health system included all patients with measured creatinine concentrations age 18 or older. ^2^We excluded counties where numbers of patients included were less than 400 patients. Unknown = unknown county of origin.

The overall prevalence of CKD was 11.8% (95% CI 11.8-12.1%) using the CKD-EPI equation and was significantly lower than estimates from the MDRD equation (13.5%, 95% CI 13.3-13.6) as shown in Table 2. To simplify exposition, all of the subsequently quoted prevalence estimates will be based on the CKD-EPI equation. Among those with CKD, the majority were classified as having Stage 3 (90.1%), with 8.0% and 1.9% classified as Stage 4 and Stage 5 respectively. The prevalence of CKD was significantly higher in women than in men (12.6% vs 10.9%, P <0.0001) and increased substantially with advancing age. Among the major counties, Leitrim had the highest prevalence (12.8%) while the prevalence in Donegal and Sligo were slightly lower at 12.0% and 11.3%. The prevalence estimates for all other counties with at least 400 or more patients are provided in Figure 1. The Additional file 1: Figure S1 provides additional information on the prevalence of CKD among participant counties along with the corresponding age distributions. The prevalence of CKD in the health system varied significantly by the presence and frequency of AKI. Compared to patients without a history of AKI, those with AKI experienced over a four-fold higher rise in CKD prevalence (10.7% versus 45.2% respectively, P <0.001).

**Table 2:** **Prevalence of chronic kidney disease (95% confidence intervals) in the Irish health system**

	CKD-EPI equation^1^	95% CI	MDRD equation^2^	95% CI
**Overall Prevalence (%)**	11.8	(11.8-12.1)	13.5	(13.3-13.6)
**By Stage (%)**				
Stage 3 CKD	10.7	(10.6-10.9)	12.5	(12.4-12.7)
Stage 4 CKD	0.95	(0.94-0.99)	0.83	(0.79-0.87)
Stage 5 CKD (No dialysis)	0.15	(0.14-0.17)	0.14	(0.12-0.15)
Stage 5 CKD (Dialysis only)^3^	0.07	(0.06-0.09)	0.07	(0.06-0.09)
**By Age group (%)** ^**4**^				
18-39	0.45	(0.39-0.51)	0.93	(0.9-1.0)
40-59	2.24	(2.13-2.35)	4.55	(4.4-4.7)
60-80	18.9	(18.6-19.3)	22.0	(21.6-22.3)
> 80	55.7	(54.9-56.4)*	52.4	(51.6-53.1)
**By Gender (%)** ^**4**^				
Women	12.6	(12.4-12.8)	15.0	(14.8-15.2)
Men	10.9	(10.7-11.1)*	11.7	(11.5-11.9)
**By County (%)** ^**4**^				
Donegal	12.0	(11.7-12.2)	13.5	(13.3-13.8)
Sligo	11.3	(11.0-11.6)	13.1	(12.7-13.4)
Leitrim	12.8	(12.3-13.3)	14.6	(14.1-15.1)
All other counties^6^	11.6	(11.1-12.0)*	13.2	(12.8,13.7)
**By Acute Kidney Injury (%)** ^**5**^				
No AKI	10.7	(10.6-10.9)	12.5	(12.3-12.6)
History of AKI	45.2	(44.3-46.1)*	45.5	(44.6-46.3)
**By Number of AKI Episodes (%)**				
0	10.9	(10.7-11)	12.4	(12.3-12.6)
1	39.1	(38.2-40)	39.5	(38.6-40.5)
2	54.8	(52.9-56.8)	54.8	(52.8-56.7)
3	64.8	(61.4-68.1)	63.4	(59.9-66.8)
4	64.1	(58.4-69.3)	65.4	(59.8-70.6)
≥5	88.7	(85.9-91.0)^*^	88.9	(86.1-91.1)

^1^eGFR CKD-EPI: Glomerular filtration rate (ml/min per 1.73 m^2^) was based on the Chronic Kidney Disease Collaborative [21].

^2^eGFR: Glomerular filtration rate (ml/min per 1.73 m^2^) was based on the Modification of Diet in Renal Disease Study Equation (MDRD) Equation [20].

^3^Patients undergoing dialysis treatment were identified from the Northwest Kidney Disease Register.

^4^Prevalence of CKD was based on the patients having stage 3 CKD or higher category.

^5^The occurrence of acute kidney injury (AKI) was based on the KDIGO Definition [16].

^*^P < 0.001 for significant differences between groups.

^6^Individual county prevalence, and associated 95% CIs, for counties aggregated into ‘Other counties’ were: Galway: 4% (2.4%, 6.6%), Derry 11% (8%,15%), Cavan: 13.5 (11.6%,15.8%) , Tyrone: 11.3% (8.2%, 15.3%), Roscommon: 10.4% (9.1%, 11.8%), Mayo: 11.7% (10.8%, 12.8%), Fermanagh 12.8% (8.5%, 18.8%), Longford: 15.8% (10.1%, 23.9%), Unknown county , 11.5% (11.0%,12.0%), Westmeath: 6.3% (0.9%, 33.5%). Note that as shown in Additional file 1: Figure S1, the age distribution of patients in Galway is significantly younger than those from other counties.

### Prevalence of detectable CKD in the Irish population

Based on census data, we estimated the prevalence of detectable CKD in the population to be 4.5% (95% CI 4.46-4.59%) as shown in Table 3. The prevalence of detectable CKD was significantly higher in women than men (5.1% versus 3.9%) and increased exponentially with advancing age. To facilitate comparison with national estimates derived from the 2007 Survey of Lifestyle, Attitudes and Nutrition (SLÁN), we recalculated prevalence but restricted to subjects age 45 and older (Table 4). The population prevalence restricted to this age group was 9.7% (95% CI 9.6-9.9%) and was higher in women than men (11.0 vs 8.4%, P < 0.001).

**Table 3:** **Prevalence of detectable CKD (95% confidence intervals) by category in the Irish population** ^**1**^

	CKD-EPI equation^2^	95% CI	MDRD equation^3^	95% CI
**Overall Prevalence (%)**	4.5	(4.46-4.59)	5.2	(5.09-5.23)
**By Stage (%)**				
Stage3	4.10	(4.04-4.16)	4.78	(4.71-4.84)
Stage 4	0.37	(0.35-0.38)	0.32	(0.31-0.34)
Stage 5 (No Dialysis)	0.06	(0.05-0.062)	0.05	(0.044-0.055)
Stage 5 (Dialysis only)	0.03	(0.025-0.035)	0.03	(0.025-0.035)
**By Age group (years) (%)**				
18-39	0.09	(0.08,0.10)	0.18	(0.17,0.20)
40-59	0.88	(0.83,0.92)	1.79	(1.73,1.86)
60-80	13.0	(12.8,13.2)	15.10	(14.85,15.36)
> 80	48.9	(48.2,49.6)^*^	46.09	(45.39-46.79)^*^
**By Gender (%)**				
Women	5.1	(5.02-5.22)	6.1	(5.98-6.19)
Men	3.9	(3.83-4.00^)*^	4.2	(4.10-4.28)^*^

^1^Population estimates were determined for 2006 and 2011 using the Irish Population census, restricted to individuals age ≥18 years of age.

^2^eGFR CKD-EPI: Glomerular filtration rate (ml/min per 1.73 m^2^) was based on the Chronic Kidney Disease Collaborative [21].

^3^eGFR: Glomerular filtration rate (ml/min per 1.73 m^2^) was based on the Modification of Diet in Renal Disease Study Equation (MDRD) Equation [20].

^*^P < 0.001 for differences in percentages between groups.

**Table 4:** **Prevalence of detectable CKD (95% confidence intervals) restricted to patients over 45**

	CKD-EPI	95% CI	MDRD	95% CI
**Prevalence based on North West Regional Database (%)** ^**1**^				
Overall	9.7	(9.6-9.9)	10.9	(10.8-11.1)
Women	11.0	(10.8-11.2)	12.9	(12.7-13.1)
Men	8.4	(8.2-8.6)*	8.9	(8.7-9.1)
**Prevalence based on SLAN Survey (%)** ^**2**^				
Overall	11.6	(9.0-14.2)	15.7	(12.7,18.7)
Women^3^	11.2	(7.3,15.2)		
Men	12.0	(9.0,14.2)		

^1^Population estimates were determined for 2006 and 2011 using the Irish Population censes, restricted to individuals age ≥18 years of age.

^2^Based on a criterion of eGFR ≤60 ml/min/1.73 m^2^.

^3^Gender stratified prevalence estimated, based on the MDRD equation were not reported in Browne et al. [18]. *P<0.001 for comparison with women.

### Period trends in CKD prevalence in the Irish Health System and General Population

**Figure 2:**
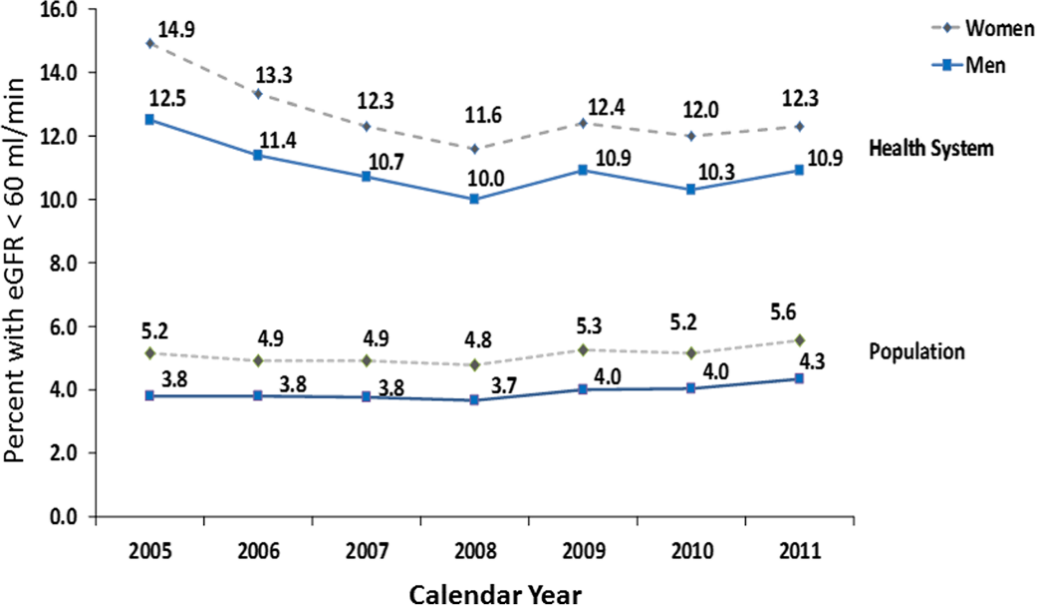
**Prevalence of detectable CKD in the adult General Population** ^**1**^ **and the Health System** ^**2**^ **for men and women from 2005-2011.** ^1^The denominator for the population prevalence was based on Irish census data for 2006 and 2011 with projected estimates for the intervening years, and restricted to adults age 18 and over. ^2^The health system included all patients with measured creatinine concentrations age 18 or older. Creatinine test results that satisfied criteria for the diagnosis of AKI based on the KDIGO criteria were excluded [16].

Period trends in prevalence of CKD within the health system are illustrated in Table 5. The prevalence peaked at 13.8% in 2005 and was lowest at 11% in 2008. In general, the prevalence fell significantly from 2005 to 2008 and remained constant thereafter. This change in trend was statistically significant (P = 0.007) when tested using Joinpoint regression analysis. This pattern was similar for men and women throughout all years, although the prevalence was significantly higher in women than in men. Within age groups, the prevalence of CKD remained relatively constant over the 7-year period with the lowest prevalence among the 18-39 year olds and the highest prevalence in the >80 year olds. Comparison of CKD prevalence within the health system and corresponding general population for men and women are also illustrated in Figure 2. The prevalence of detectable CKD in the general population fell significantly from 4.5 to 4.2% between 2005 and 2008 (P = 0.018), and increased thereafter to 5.0 in 2011 (Figure 2).

**Table 5:** **Period trends in CKD prevalence in the health system** ^**1**^

	Entire cohort	2005	2006	2007	2008	2009	2010	2011
**Count (n)**	206,729	64,674	71,118	75,956	79,300	81,338	85,200	89,679
**Overall Prevalence**	11.9	13.8	12.4	11.6	10.9	11.8	11.4	11.8
**By stage**								
3	10.73	12.4	11.2	10.5	9.8	10.7	10.3	10.7
4	0.95	1.18	1.09	0.96	0.87	0.87	0.83	0.88
5	0.15	0.26	0.18	0.16	0.15	0.13	0.11	0.12
**By Gender**								
Women	12.7	14.9	13.3	12.3	11.6	12.4	12.0	12.3
Men	10.9	12.5	11.4	10.7	10.0	10.9	10.3	10.9
**By Age group**								
18-39	0.4	0.6	0.5	0.5	0.4	0.5	0.3	0.4
40-59	2.2	2.9	2.5	2.2	2.1	2.2	2.0	2.0
60-80	18.9	22.8	20.4	18.8	17.1	18.6	17.3	18.3
> 80	55.6	58.6	56.5	54.4	52.5	56.3	55.5	56.0
**By age group (females)**								
18-39	0.35	0.48	0.36	0.32	0.33	0.41	0.33	0.25
40-59	2.18	2.8	2.5	2.2	2.0	2.0	1.9	2.0
60-80	20.40	24.9	22.1	20.2	18.5	20.2	18.6	19.6
> 80	57.63	60.7	57.9	56.6	54.5	58.2	57.6	58.3
**By age group (males)**								
18-39	0.6	0.6	0.6	0.8	0.6	0.6	0.4	0.6
40-59	2.3	2.9	2.5	2.2	2.2	2.4	2.1	2.1
60-80	17.4	20.6	18.7	17.4	15.7	17.0	16.1	17.0
> 80	52.4	55.3	54.1	50.8	49.4	53.3	52.2	52.4
**By Geographic origin**								
Sligo	11.3	13.6	12.0	11.1	10.6	10.7	10.5	11.0
Donegal	12.0	13.5	12.4	11.7	10.8	12.2	11.5	12.0
Leitrim	12.8	15.4	13.5	12.1	12.0	12.8	12.3	12.4
All other counties	11.6	13.9	12.5	11.4	10.7	10.8	10.3	11.1
**By Acute Kidney Injury** ^**2**^								
No AKI	10.7	12.6	11.2	10.4	9.8	10.7	10.3	10.7
With AKI	45.2	50.8	45.5	45.6	44.3	42.4	44.3	44.6

^1^Estimated Glomerular filtration rate (ml/min per 1.73 m^2^) was determined by the CKD-EPI formula [21].

^2^The occurrence of acute kidney injury (AKI) was based on the KDIGO Definition [16].

### Factors associated with CKD prevalence in the Health System

Table 6 describes the relationship of demographic, clinical and geographic factors with CKD prevalence within the health system. In this series of analysis, we explored associations of each listed covariate with CKD presence in demographic–adjusted and fully adjusted models. The prevalence of CKD correlated with advancing age and female gender. CKD was more likely to be detected when screening in an outpatient department (Adjusted odds ratio (AOR) 1.50, 95% CI 1.44-1.57) or community health facilities (AOR 1.09, 1.03-1.15) and less likely to found when screening in emergency departments (AOR, 0.90, 0.87-0.94) or inpatient wards (AOR 0.87, 0.83-0.91). Compared to patients entering the health system in 2005, those that were tested in subsequent years were less likely to have CKD, P < 0.001. The likelihood of CKD varied significantly by county of residence. Compared to patients resident in Sligo (referent group, AOR = 1.00), patients resident in Donegal (AOR = 1.07, 1.02-1.11) and Leitrim (AOR = 1.09, 1.03-1.16) were significantly more likely to have CKD. The relationship between AKI and CKD was significant and substantial. Compared to patients without any prior episode of AKI during the calendar period, each episode of AKI conferred an increasing likelihood of CKD. For example, patients who had incurred three AKI events experienced a 7.4 fold higher likelihood of CKD compared with those who had no prior AKI event. The C-statistic of the model was 0.86 indicating excellent discrimination.

**Table 6:** **Factors associated with CKD prevalence in the Irish health system** ^**1**^

Variable	Odds ratio	95% CI	P-value	Odds ratio	95% CI	P-value
	Adjusted demographics^2^			Adjusted all factors^3^		
**Demographic factors**						
**Age group (years)**						
18-39	1.00 (referent)			1.00 (referent)		
40-59	5.0	(4.32,5.78)	<.0001	5.14	(4.46,5.92)	<.0001
60-79	50.27	(43.74,57.79)	<.0001	51.17	(44.68,58.59)	<.0001
> = 80	265.74	(230.83,305.88)	<.0001	260.66	(227.26,298.93)	<.0001
**Sex**						
Women (vs. men)	1.17	(1.13,1.22)	<.0001	1.21	(1.17,1.26)	<.0001
**Location of supervision**						
General Practice (GP)	1.00 (referent)			1.00 (referent)		
Emergency room vs. GP	1.04	(1.01,1.08)	0.01	0.90	(0.87,0.94)	<.0001
Inpatient location vs. GP	1.17	(1.12,1.23)	< .0001	0.87	(0.83,0.91)	<.0001
Outpatient Department vs. GP	1.64	(1.58,1.72)	< .0001	1.50	(1.44,1.57)	<.0001
Outside facility vs. GP	1.13	(1.06,1.19)	< .0001	1.09	(1.03,1.15)	0.004
**Calendar Year**						
2005	1.00 (referent)			1.00 (referent)		
2006 vs. 2005	0.88	(0.85,0.90)	< .0001	0.87	(0.84,0.89)	<.0001
2007 vs. 2005	0.79	(0.76,0.81)	< .0001	0.79	(0.76,0.81)	<.0001
2008 vs. 2005	0.72	(0.69,0.74)	< .0001	0.71	(0.68,0.73)	<.0001
2009 vs. 2005	0.80	(0.77,0.82)	< .0001	0.78	(0.76,0.81)	<.0001
2010 vs. 2005	0.74	(0.72,0.76)	< .0001	0.73	(0.71,0.76)	<.0001
2011 vs. 2005	0.78	(0.76,0.80)	< .0001	0.77	(0.74,0.79)	<.0001
**# Acute Kidney Injury** ^**4**^ **episodes**						
0	1.00 (referent)			1.00 (referent)		
1 vs. 0	2.36	(2.24,2.48)	< .0001	2.41	(2.29,2.54)	<.0001
2 vs. 0	4.11	(3.71,4.54)	< .0001	4.20	(3.79,4.65)	<.0001
3 vs. 0	7.19	(5.90,8.77)	< .0001	7.39	(6.06,9.02)	<.0001
4 vs. 0	9.44	(6.74,13.1)	< .0001	9.70	(6.94,13.55)	<.0001
5 vs. 0	120.0	(79.7,180.6)	< .0001	102.56	(69.15,152.09)	<.0001
**County of Residence**						
Sligo	1.00 (referent)			1.00 (referent)		
Donegal vs. Sligo	1.05	(1.01,1.10)	0.02	1.07	(1.02,1.11)	0.0035
Leitrim vs. Sligo	1.08	(1.02,1.14)	0.01	1.09	(1.03,1.16)	0.0031
Other counties vs. Sligo	1.03	(0.97,1.09)	0.30	1.07	(1.01,1.13)	0.0244
**Primary Hospital** ^**5**^						
Letterkenny vs. Sligo^6^	1.00	(0.96,1.03)	0.88			

^1^Estimated Glomerular filtration rate (ml/min per 1.73 m^2^) calculated by the eGFR CKD-EPI formula [21] was used to calculate prevalence.

^2^Adjusted for age, sex only.

^3^Adjusted for age, sex, provider location, calendar year, county of residence, acute kidney injury.

^4^The occurrence of acute kidney injury (AKI) was based on the KDIGO Definition [16].

^5^Primary hospital location was not included in the final multivariable model.

^6^Letterkenny General Hospital is located in county Donegal, while Sligo General Hospital is the primary hospital for the county of Sligo.

## Discussion

In this surveillance study, we sought to describe the prevalence and variation of CKD in the Irish health system. We found that the overall prevalence of CKD in the health system was 11.8% (approximately 1 in 8 patients), higher in women than men, and increased substantially with advancing age. There was evidence for regional variation in CKD prevalence that was not explained by differences in age or sex distributions. We also found that most CKD could be easily identified from designated locations within the health system; the inpatient ward, outpatient clinic and the community hospital. Given the emerging interest in the AKI-CKD link [17, 25, 26], we demonstrated that among patients with a prior history of AKI, the prevalence of CKD was almost 50%, and that the likelihood of CKD increased with increasing frequency of AKI events. At the population level, we showed that the prevalence of detectable CKD in the region was 4.5%, and that when restricted to patients age 45 and older, our estimate of CKD prevalence was similar to that derived from the nationally representative SLAN survey.

The present study provides compelling evidence that passive surveillance of CKD within a regional health system is achievable and provides accurate and essential information on disease burden and its determinants that are necessary for service planning and resource allocation. Recognising the global challenge of CKD and its adverse complications, there is an urgent need for well-developed surveillance systems to capture CKD and track clinical outcomes within a countries health system [14]. Through extraction of data from the regional laboratory system and administrative systems we were able to estimate the prevalence of CKD, identify important correlates and assess prevalent trends from 2005-2011. This initiative is the first of its kind in Ireland to support systematic surveillance of CKD and provides the foundation stone for a national surveillance programme.

The current study is the largest conducted in Ireland and provides a reliable estimate of CKD prevalence in the Irish health system. Our sample of 206, 729 patients, included all adults within the northwest health system with at least one measured creatinine concentration. Our prevalence estimate of 11.8% was lower than that reported by Glynn *et al* who found a prevalence of 16.7% CKD among 2,602 primary care patients age >50 years and Anderson *et al* who reported an even higher prevalence of 20% among elderly patients with established cardiovascular disease [27, 28]. However, unlike Glynn et *al,* we did not restrict our sample to high-risk older age groups. It is possible that differences in CKD prevalence reflect differences in definitions of CKD, the choice of sampling frame, the measure used for its determination, and the size of the denominator. Systematic reviews of prevalence studies have found substantial variation in estimates ranging from 0.6-42.6% [29]. To facilitate comparisons with national data, we estimated the population prevalence of detectable CKD in the northwest region by substituting the population in the health system with that of the general population as our new denominator. The derived population estimate of 4.5% was significantly lower than reported by US and other countries, but remarkably similar to that generated from Scottish and UK national data [1, 15, 30]. Moreover, when we compared our data with national data from the SLAN survey using similar definitions (i.e. limited to age 45 and over), our estimate of 9.7% was slightly lower but nonetheless similar to the 11.6% that reported by Browne et al [18]. These data would suggest that a large proportion of CKD within the general population is already captured within the Irish health system.

The availability of serial measurements on serum creatinine concentrations over time allowed us a unique opportunity to capture the presence and frequency of AKI in the health system and explore association with CKD presence. When modeled as a binary variable or in categories, AKI was significantly and independently associated with CKD. Furthermore, our multivariable model demonstrated a steep rise in gradient of risk with each episode of AKI. These observations support the increasing body of evidence that link AKI with risk of CKD [17, 25, 26]. Chawla *et al* found higher rates of disease progression to stage 4 CKD among AKI survivors in a population of US veterans [17]. Similarly, studies by Ishani et al and Thaker *et al* have identified strong associations between the severity of AKI and risk of CKD progression in diverse populations [25, 26]. The evidence thus far would suggest that the frequency and severity of AKI in the health system has a direct impact on the burden of CKD in the population. One might therefore hypothesize that preventive efforts to reduce the frequency of AKI, in combination with improved management of the usual CKD risk factors such as diabetes, may lead to a stabilization of CKD prevalence and eventually a possible decline.

Our study is the first to shed new light on the frequency of testing for CKD in the Irish health system, and the relationship of geographic location and location of supervision with CKD. Overall, the majority of blood tests for assessment of kidney function were ordered by general practitioners (67.8%) followed by emergency room physicians (15.1%). As in many national health systems, the primary care provider plays a pivotal role in chronic disease management both in screening for and monitoring of CKD in the health system [31, 32]. There were 1, 388, 625 creatinine tests ordered for 206, 729 patients, giving an average of 6.7 tests per patient. Based on these data, one could infer that each patient had on average 6.7 opportunities to detect the presence of impaired kidney function. CKD was more likely to be present if blood tests were ordered at the outpatient department, an inpatient admission, outside community hospital and to a lesser degree the emergency room compared to general practice. Blood tests ordered through the outpatient department had the strongest associations with CKD. These data reflect the fact that patients who have their serum creatinine measured are likely to interact with the health system at different locations, each providing an opportunity to screen for and monitor CKD. In this analysis, we also found differences in the prevalence of CKD by county of residence. Although the crude prevalence did not vary substantially, we did identify differences when adjustments were made for age and sex. Differences in the testing rate, underlying risk factors and their treatment may be responsible for these differences.

There are limitations to the present study. Our definition of CKD was based on creatinine- derived GFR estimating equations alone and would have benefited from inclusion of data on albuminuria in order to detect earlier stages of disease. We also acknowledge that our study lacked data on major risk factors for CKD including diabetes and hypertension which would have strengthened our analysis. Notwithstanding these deficits, our study had several major strengths. First, our estimate of kidney function was based on original standardised creatinine concentrations from which we derived estimated eGFR values using the CKD-EPI and MDRD equations and were thus not reliant on administrative claims data for CKD diagnosis. Second, we had complete access to the regional laboratory information system in the Northwest Region which included clinical data from all primary care and secondary care providers providing excellent generalizability. Third, our study was conducted over a 7-year period and we were able to provide information on period trends. Fourth, the size of the population was extremely large thereby allowing us generate very precise estimates of CKD across representative subgroups.

## Conclusions

The current study is the largest study to-date to describe the prevalence of CKD in the Irish Health system and provide comparisons with national data. It demonstrates that the detection of CKD within the health system is remarkably similar to percentages reported within the general Irish population. It highlights significant variation in prevalence across representative groups and provides compelling evidence that AKI is strongly linked with CKD burden. Finally, while the burden of CKD has declined from 2005-2008, our analysis suggests a more recent trend of increasing growth which has important clinical and economic consequences. This study is the first output of National Kidney Disease Surveillance System in Ireland, a programme that will serve as a major resource for tracking and monitoring kidney disease and outcomes in the Irish health system.

## Electronic supplementary material

Additional file 1: Figure S1: Age distribution of each County within the Health System, number of residents in each county and the prevalence of CKD for each of 12 counties and Unknown county of origin (with 95% Confidence Intervals). ^2^The health system included all patients with measured creatinine concentrations age 18 or older. Creatinine test results that satisfied criteria for the diagnosis of AKI based on the KDIGO criteria were excluded [16]. (TIFF 5 MB)
