# Supplementary figures and images for: Prevalence and variation of Chronic Kidney Disease in the Irish health system: initial findings from the National Kidney Disease Surveillance Programme
Source: BMC Nephrol. 2014 Nov 25;15:185. doi: 10.1186/1471-2369-15-185 (PMC4258258; doi:10.1186/1471-2369-15-185)

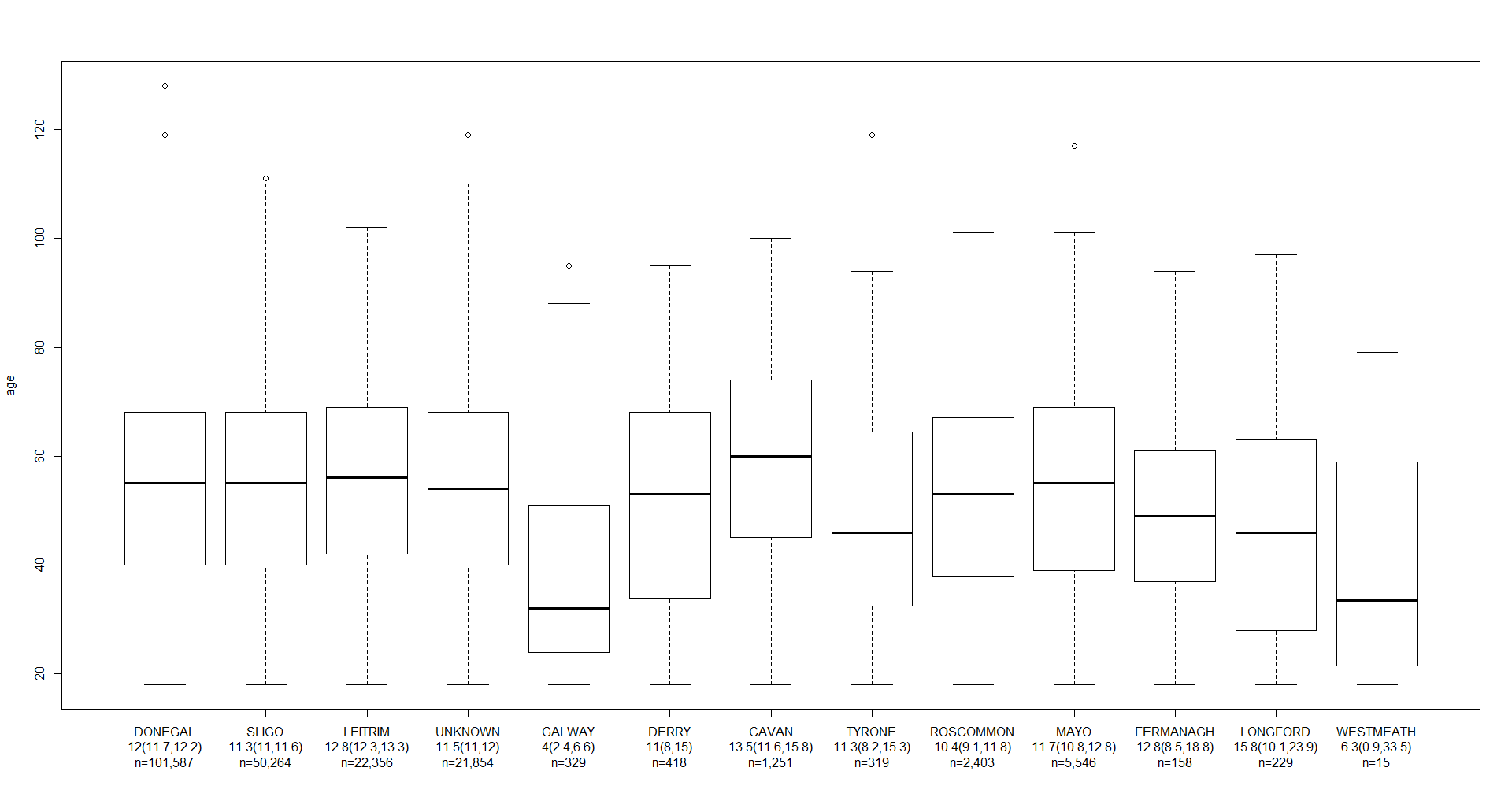

Supplement: Supplementary file 1 — Additional file 1: Figure S1: Age distribution of each County within the Health System, number of residents in each county and the prevalence of CKD for each of 12 counties and Unknown county of origin (with 95% Confidence Intervals). 2The health system included all patients with measured creatinine concentrations age 18 or older. Creatinine test results that satisfied criteria for the diagnosis of AKI based on the KDIGO criteria were excluded [16]. (TIFF 5 MB) [file 12882_2014_875_MOESM1_ESM.tiff]
